# Cardiac magnetic resonance T1 mapping predicts outcome in patients with a systemic right ventricle: *a sub-analysis of the SERVE study*

**DOI:** 10.1093/ehjopen/oeag138

**Published:** 2026-09-08

**Authors:** Odile Burdet, Kerstin Wustmann, Barbara E U Burkhardt, Emanuela R Valsangiacomo Buechel, Jean-Paul Vallée, Judith Bouchardy, Matthias Greutmann, Markus Schwerzmann, Harald Gabriel, Dominik Stambach, Daniel Tobler, Juerg Schwitter, Tobias Rutz

**Affiliations:** Service of Cardiology, Lausanne University Hospital and University of Lausanne, Rue du Bugnon 46, 1011 Lausanne, Switzerland; Department of Paediatric and Congenital Cardiology, German Heart Center Munich, Lazarettstrasse 36, 80636 Munich, Germany; Department of Pediatric Cardiology, University Children’s Hospital Zürich, Lengstrasse 30, 8008 Zürich, Switzerland; Department of Pediatric Cardiology, University Children’s Hospital Zürich, Lengstrasse 30, 8008 Zürich, Switzerland; Division of Radiology, Geneva University Hospitals and University of Geneva, Rue Gabrielle-Perret-Gentil 4, 1205 Geneva, Switzerland; Service of Cardiology, Lausanne University Hospital and University of Lausanne, Rue du Bugnon 46, 1011 Lausanne, Switzerland; Division of Cardiology, Geneva University Hospitals and University of Geneva, Rue Gabrielle-Perret-Gentil 4, 1205 Geneva, Switzerland; University Heart Center, Department of Cardiology, University Hospital of Zurich, Rämistrasse 100, 8091 Zurich, Switzerland; Center for Congenital Heart Disease, University Hospital Inselspital, University of Bern, Freiburgstrasse 20, 3010 Bern, Switzerland; Division of Cardiology, University of Ottawa Heart Institute, Ruskin Street 40, Ottawa, ON K1Y 4W7, Canada; Medical University of Vienna, Department of Cardiology, Adult Congenital Heart Disease Program, Währinger Gürtel 18-20, 1090 Vienna, Austria; Paediatric Cardiology, Ostschweizer Kinderspital, Spitalstrasse 10, 9006 St. Gallen, Switzerland; University Heart Center, University Hospital Basel, University of Basel, Petersgraben 4, 4031 Basel, Switzerland; Service of Cardiology, Lausanne University Hospital and University of Lausanne, Rue du Bugnon 46, 1011 Lausanne, Switzerland; Service of Cardiology, Lausanne University Hospital and University of Lausanne, Rue du Bugnon 46, 1011 Lausanne, Switzerland

**Keywords:** Systemic right ventricles, T1 mapping, ECV, Congenital heart disease, CMR

## Abstract

**Aims:**

In patients with a systemic right ventricle (sRV), the sRV is faced with an increased afterload compared to a right ventricle in sub-pulmonary position, leading to the development of fibrosis followed by ventricular dysfunction. Cardiac magnetic resonance (CMR) T1 mapping (T1) and its derived parameter, such as the extracellular volume (ECV), allow quantification of interstitial fibrosis. The SERVE study, a multicentric, placebo-controlled trial evaluated the effect of tadalafil in sRV patients. The aim of this sub-analysis of the SERVE trial was to evaluate (i) the relationship between T1 mapping parameters and the clinical composite endpoint of hospitalization for heart failure, clinically relevant arrhythmias, and all-cause mortality and (ii) their evolution over time, their correlation with CMR functional parameters, and the impact of study treatment.

**Methods and results:**

Patients included in the SERVE trial underwent a 1.5T CMR at baseline and at 1- and 3-year follow-up. For each CMR exam, native and post-contrast T1, as well as ECV, were calculated. In addition, late gadolinium enhancement (LGE) imaging was performed. Cardiac magnetic resonance was available for 60 patients. Native sRV T1 and ECV above the 75th percentile, but not LGE, were associated with the clinical composite endpoint. There was no significant change and no influence of tadalafil treatment on sRV T1 and ECV.

**Conclusion:**

T1 mapping as a non-invasive biomarker of the degree of myocardial fibrosis predicts outcome in patients with sRV and should be incorporated in risk stratification in these patients. The phosphodiesterase-5 inhibitor tadalafil appears to have no impact on the development of interstitial or macroscopic fibrosis.

## Graphical Abstract

**Graphical Abstract:**
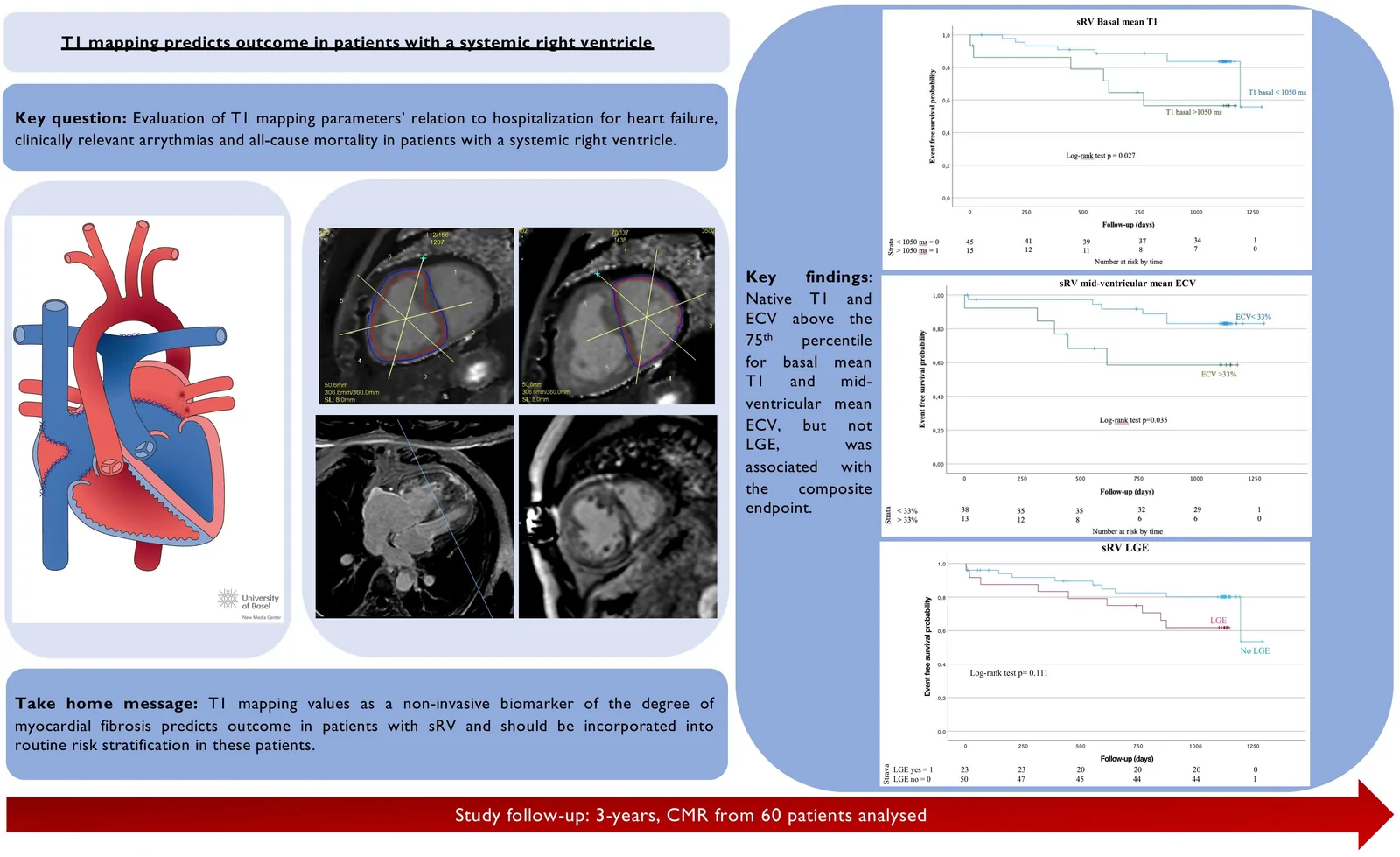

## Introduction

Patients with congenital heart disease (CHD) and a systemic right ventricle (sRV) comprise patients with d-transposition of the great arteries (d-TGA) after atrial switch procedures or patients with congenitally corrected transposition of the great arteries (ccTGA). These patients are at high risk of heart failure.^1^ Given its position in the systemic circulation, the sRV of these patients is exposed to systemic pressures and hence to increased afterload^2^ compared to a right ventricle in sub-pulmonary position. This unique physiology induces cardiac remodelling characterized by ventricular hypertrophy and dilatation associated with the development of myocardial fibrosis.^3–7^ Fibrosis has been shown to be the underlying pathological basis for a variety of cardiomyopathies and has multiple consequences, leading to altered ventricular function associated with increased morbidity and mortality.^1,3–6^

Cardiac magnetic resonance (CMR) imaging has become an important imaging modality in CHD, particularly in patients with sRV.^8^ It allows non-invasive, precise, reproducible, and radiation-free analysis of myocardial structure and quantification of myocardial tissue composition.^9^ The widely used ‘late gadolinium enhancement’ (LGE) CMR technique is considered the gold standard for the identification of macroscopic fibrosis.^10^ However, in a variety of non-ischaemic cardiomyopathies, such as CHD, the fibrotic process is often characterized by diffuse interstitial, i.e. microscopic, myocardial fibrosis.^11–13^ Cardiac magnetic resonance T1 mapping (T1) technique allowsidentification of interstitial myocardial fibrosis by determining T1 values for each voxel of the image and calculating the extracellular volume (ECV).^3^ These parameters are validated as robust measures of myocardial interstitial fibrosis, as they correlate with a higher collagen content of the myocardium.^14–16^

The benefit of heart failure therapy has been well demonstrated in patients with acquired left ventricular failure, but similar effects could not be shown for patients with heart failure related to a sRV.^10–19^ As a new therapeutic option, studies have demonstrated promising results regarding phosphodiesterase-5 (PDE-5) inhibition in hypertrophic RV.^20,21^

The SERVE (Effect of Phosphodiesterase-5 Inhibition With Tadalafil on Systemic Right Ventricular Size and Function) trial was a multi-centre, double-blind, randomized, placebo-controlled clinical trial designed to assess the effects of tadalafil on sRV over time.^1,21^

The present study is a pre-defined sub-analysis of the SERVE trial and prospectively evaluates the clinical relevance of interstitial myocardial fibrosis determination in patients with a sRV using the T1 mapping technique.

### Study objectives

The main objective was to evaluate if T1 values reflecting diffuse interstitial fibrosis or LGE values reflecting regional fibrosis could help to predict outcome. In line with the main study, outcome was a composite of hospitalization for heart failure, clinically relevant arrhythmias, and all-cause mortality.^1,21^

The secondary objectives were to assess the impact of the study medication on the evolution of the degree of fibrosis in the myocardium during follow-up and to evaluate how T1 mapping values were related to CMR functional parameters.

The primary hypothesis is that myocardial fibrosis reflected by increased T1 mapping values and the presence of LGE is associated with worse clinical outcomes.

The secondary hypothesis is that, in line with the primary hypothesis of the SERVE trial, treatment with tadalafil compared with placebo is associated with reduced microscopic myocardial fibrosis during follow-up and more favourable CMR parameters.

## Methods

### Patient selection and randomization

Details of the study design of the SERVE trial have been published previously [The main study is registered at ClinicalTrials.gov (Identifier: NCT03049540)].^1,21^ In brief, the SERVE study is a double-blind, randomized, placebo-controlled, multi-centre superiority trial comparing treatment with tadalafil vs. placebo in a 1:1 ratio, over a 3-year follow-up (study period 2017–2021). A total of 100 clinically stable adult patients [mean age 39 (25–75) years] with d-TGA after atrial switch operation (75%) or with ccTGA (25%) were included, of whom 51 were randomly assigned to the tadalafil and 49 to the placebo group after baseline examinations.

The study complies with the Declaration of Helsinki. The responsible ethics committees and the competent authorities approved the protocol. All participants gave written informed consent prior to participation in the study.

All patients underwent clinical examination, standardized transthoracic echocardiography, CMR, or computed tomography (CT) in patients with contraindications for CMR.

### Cardiac magnetic resonance protocol

Cardiac magnetic resonance was performed in patients without contraindications at baseline and at the 1-year and 3-year follow-ups with a standardized acquisition protocol.

Electrocardiogram- (ECG) gated CMR imaging was performed using 1.5 Tesla scanners (Siemens and GE Healthcare) with a 32-channel phased-array surface receiver coil. Cine images were acquired using a breath-hold steady-state free precession sequence technique in short-axis views (8 mm slices without gap, 10–15 slices). For pre-contrast T1 mapping, an ECG-triggered modified Look-Locker inversion recovery (MOLLI) sequence [using the scheme 3(3)3(3)5] on basal and mid-ventricular short-axis slices was used with an inplane spatial resolution of <2 mm. Ten minutes after the intravenous bolus administration of a 0.2 mmol/kg gadolinium-based contrast agent, LGE images were acquired using a 2D breath-hold phase-sensitive segmented inversion-recovery gradient echo pulse sequence in the same orientations as cine images. Inversion time was individually optimized to null normal myocardium. Post-contrast T1 mapping images were acquired following LGE imaging (typically 20 min after contrast bolus injection) using a MOLLI sequence [4(1)3(1)2 scheme]. Ventricular volumes and ejection fractions were determined as previously described.^1,21^

### T1 mapping analysis

Pre- and post-contrast T1 mapping images were first visually reviewed for adequate image quality. The exclusion criteria for images included mistriggering, folding artefacts, and incomplete coverage. All images were carefully reviewed according to these criteria. Myocardial T1 relaxation times (T1) were measured using the GTVolume (TM) software (GyroTools LLC, Zurich, Switzerland), by readers O.B. and T.R.

After manual tracing of the endocardial and epicardial borders, the basal and mid-ventricular right ventricular (RV) slices were divided into six equal segments and the left ventricular (LV) into four equal segments (cf. *Figure 1*). T1 values were calculated for each individual segment. The septal segments were attributed to the RV. For each subject, native and post-contrast T1 values were first calculated at the segmental level for each individual segment and then a global ‘mean T1’ value was derived by averaging the T1 values of all analysed segments within the RV and LV, respectively. No weighting was applied; all segments contributed equally to the calculation of the mean T1 (*Figure 1*).

**Figure 1:**
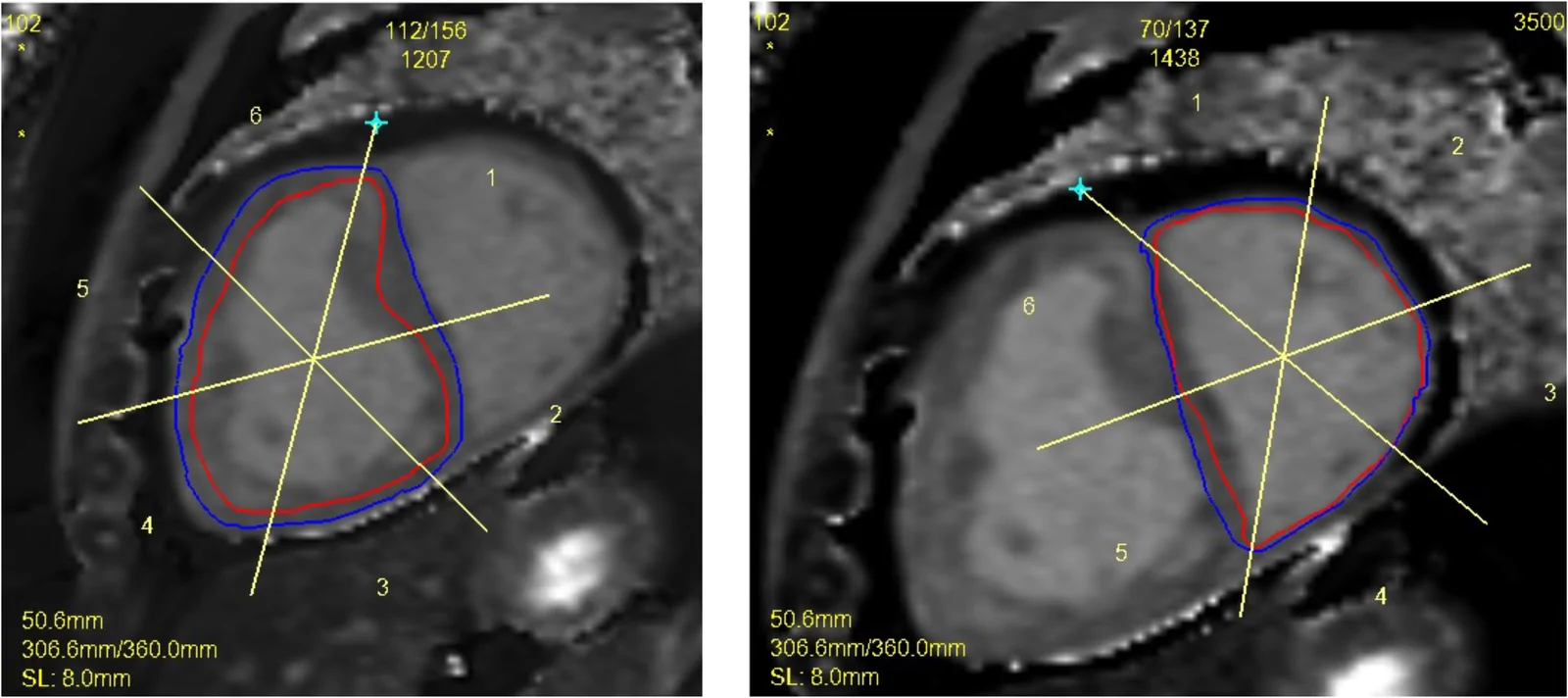
T1 mapping and segmentation. After manual tracing of the endocardial and epicardial borders, the basal and mid-ventricular right ventricular (RV) slices were divided into six equal segments and the left ventricle into four equal segments. Mean T1 values were calculated for each individual segment. The septal segments were attributed to the RV.

Extracellular volume was calculated for each segment. It reflects the proportion of myocardial tissue occupied by the extracellular space and is calculated by using pre- and post-contrast T1mapping values and blood pool, corrected by the haematocrit using this formula^10^:


$$ECV=(1-hematocrit)\;\frac{\frac{1}{post\;contrast\;T1\;myo}-\frac{1}{native\;T1\;myo}}{\frac{1}{post\;contrast\;T1\;blood}-\frac{1}{native\;T1\;blood}}$$

Exclusion criteria comprised segments with a wall thickness < 2 pixels or discordant values (outliers, defined as values > 20% larger than neighbouring segments). Intra- (O.B.) and inter-observer (T.R.) variabilities were obtained based on repeated measurements in ten randomly chosen patients.

### Late gadolinium enhancement analyses

Late gadolinium enhancement was evaluated by visual analysis by one observer (KW). Late gadolinium enhancement was considered present if a hyperintense signal was identifiable in two views in the presence of correct nulling and no artefact (*Graphical abstract*). Late gadolinium enhancement was evaluated on short-axis planes including compact myocardium, trabeculations, and papillary muscles, and RV–LV insertion regions. The septum was considered part of the RV.

### Statistical analysis

Continuous variables are indicated as mean and standard deviation or median and range as appropriate. Normality was tested using the Shapiro–Wilk test. Categorical variables are indicated as numbers and percentages. Bland–Altman analyses were used to assess intra- and inter-observer variability. To evaluate the influence of T1 and ECV on functional parameters, patients were divided into two groups with a cut-off value corresponding to the 75th percentile. An independent *t*-test was used to compare T1 and ECV values between groups with and without adverse events during follow-up, and the χ^2^ test was used to compare LGE values between these same groups. Kaplan–Meier analyses were used to compare the occurrence of the composite primary outcome between groups with values above or below the determined calculated cut-off corresponding to the 75th percentile of native T1 and ECV values. In case of multiple events, the event-free survival time from the date of inclusion until the occurrence of the first event was considered. Statistical significance for the difference between groups was assessed by means of the log-rank test. To compare the evolution of T1 values during follow-up between placebo and treatment groups, the two-way mixed analysis of variance (ANOVA) with Bonferroni *post hoc* analysis was used. Pearson’s correlation was used to assess correlations between functional parameters and native T1 and ECV values of the RV.

Cox proportional survival regression analyses were performed to determine predictors of the clinical outcome.

Statistical analysis was performed using SPSS version 28.0.1.1 (IBM Corp., Chicago, USA) and Excel version 16.52 (Microsoft, Redmond, USA).

## Results

### Study population

Of the 100 patients included in the SERVE study, 78 underwent CMR exams and 22 underwent cardiac CT at inclusion. T1 mapping was available for 60 patients at baseline, 62 at 1-year follow-up, and 61 at 3-year follow-up. In 18 patients, T1 mapping images were not available due to absent acquisition or insufficient imaging quality not allowing the analysis of any segments. Late gadolinium enhancement was available for 75 patients at baseline and 60 patients at 3-year follow-up. *Table 1* shows RV and LV functional parameters at baseline.

**Table 1:** Functional parameters at baseline

		Placebo	Treatment	*P* value
		*n* = 49	*n* = 51	
d-TGA		45	47	
ccTGA		4	4	
Sex	*Women*	19	14	0.229
Age	*Years*	40 ± 11	41 ± 10	0.587
Weight	*kg*	75 ± 15	79 ± 11	0.084
Height	*cm*	171 ± 9	174 ± 7	0.094
BMI	*kg/m^2^*	25 (19–40)	26 ± 4	0.085
BSA	*m^2^*	1.85 ± 0.03	1.95 ± 0.02	0.017
Haematocrit	*%*	43 ± 3.7 (*n* = 48)	44 ± 3 (*n* = 50)	0.259

Baseline CMR RV functional parameters				
		*n* = 40	*n* = 38	*P* value
End-diastolic volume	*mL*	217 (116–429)	243 ± 59	0.061
End-diastolic volume indexed	*mL/m^2^*	118 (73–244)	125 ± 30	0.337
End-systolic volume indexed	*mL/m^2^*	64 (36–178)	71 (37–151)	0.243
RV mass	*g*	87 ± 22	94 ± 21	0.186
Ejection fraction	*%*	46 ± 7	44 ± 7	0.153


Baseline CMR LV functional parameters				
		*n* = 40	*n* = 38	*P* value
End-diastolic volume	*mL*	140 ± 38	142 (95–223)	0.793
End-diastolic volume indexed	*mL*	76 ± 21	73 (48–122)	0.486
End-systolic volume indexed	*mL*	26 ± 9	26 ± 8	0.927
Ejection fraction	*%*	66 ± 6	65 ± 5	0.405
		*n* = 49	*n* = 51	
Adverse events total		12 (= 24%)	15 (= 29%)	0.579
Hospitalization for heart failure		10 (=20%)	12 (=24%)	0.706
Arrhythmia		8 (=16%)	10 (=20%)	0.669
All-cause mortality		1 (2%)	1 (2%)	0.977
LGE		11/40 (= 27%)	13/35 (= 37%)	0.372

### T1 mapping for prediction of outcome

Out of the 60 patients included in our study, there was a total of 12 adverse events (24%) in the placebo group and 15 (30%) in the treatment group: these included hospitalization for heart failure [10 (20%) in the placebo group, 12 (24%) in the treatment group]; clinically relevant arrhythmias [8 (16%) in the placebo group and 10 (20%) in the treatment group]; and all-cause mortality (one in each group).

Kaplan–Meier analysis revealed a higher incidence of adverse events in patients with native T1 and ECV values above the pre-defined cut-off for RV native T1 at basal (P75 = 1050 ms) and for ECV at the mid-ventricular level (P75 = 33%). Values of RV native mid-ventricular T1 and RV basal ECV showed similar trends, although not statistically significant (*Figure 2*). Supplementary Table 1 shows the comparison of native T1 and ECV between groups of patients with and without adverse events (Supplementary material online, *Table S1*).

**Figure 2:**
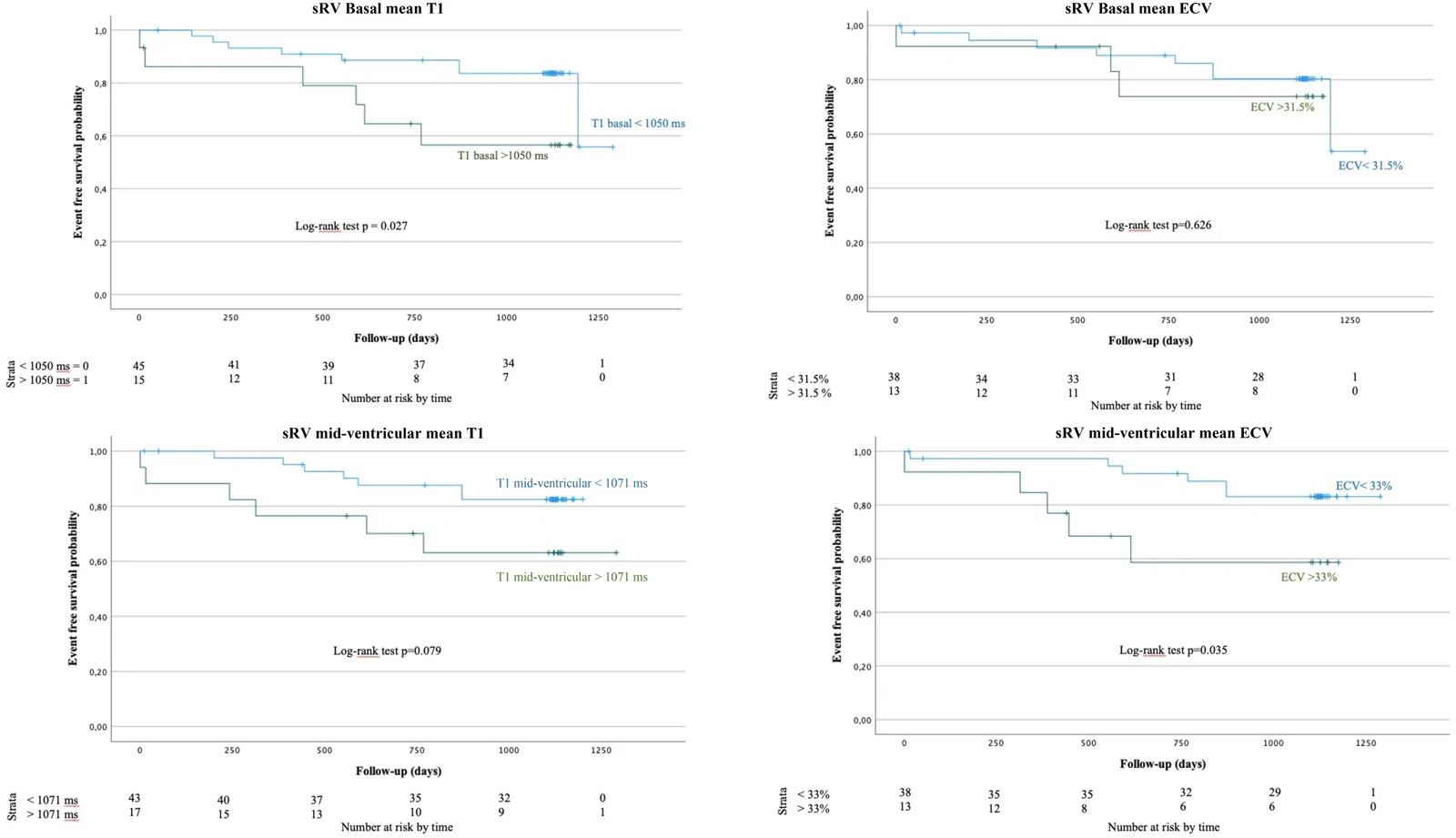
Systemic right ventricular T1 (T1) and extracellular volume (ECV) values for prediction of outcome. Kaplan–Meier analysis revealed a higher incidence of adverse events in patients with native T1 and ECV values above the pre-defined cut-off for right ventricular (RV) native T1 at basal (P75 = 1050 ms) and for ECV at the mid-ventricular level (P75 = 33%). Values of RV native mid-ventricular T1 and RV basal ECV showed similar trends, although not statistically significant.

### Late gadolinium enhancement analysis and clinical outcome

Almost all patients (98%) had LGE of at least either the superior or inferior insertion point or both. Therefore, insertion point LGE was not considered for analyses. Late gadolinium enhancement was not significantly associated with adverse outcomes (*Figure 3,*  Supplementary material online, *Table S2*), although the presence of LGE tended to be associated with worse functional parameters and more adverse events (see Supplementary material online, *Table S5*). Late gadolinium enhancement within the RV wall (excluding insertion point LGE) was present in 27% of patients in the placebo group and 37% in the treatment group (*P* = 0.798). After pooling both groups, 37% adverse events were observed in the group of patients with LGE within the RV wall vs. 19% adverse events in patients without LGE within the RV wall (*P* = 0.097; see Supplementary material online, *Table S3*).

**Figure 3:**
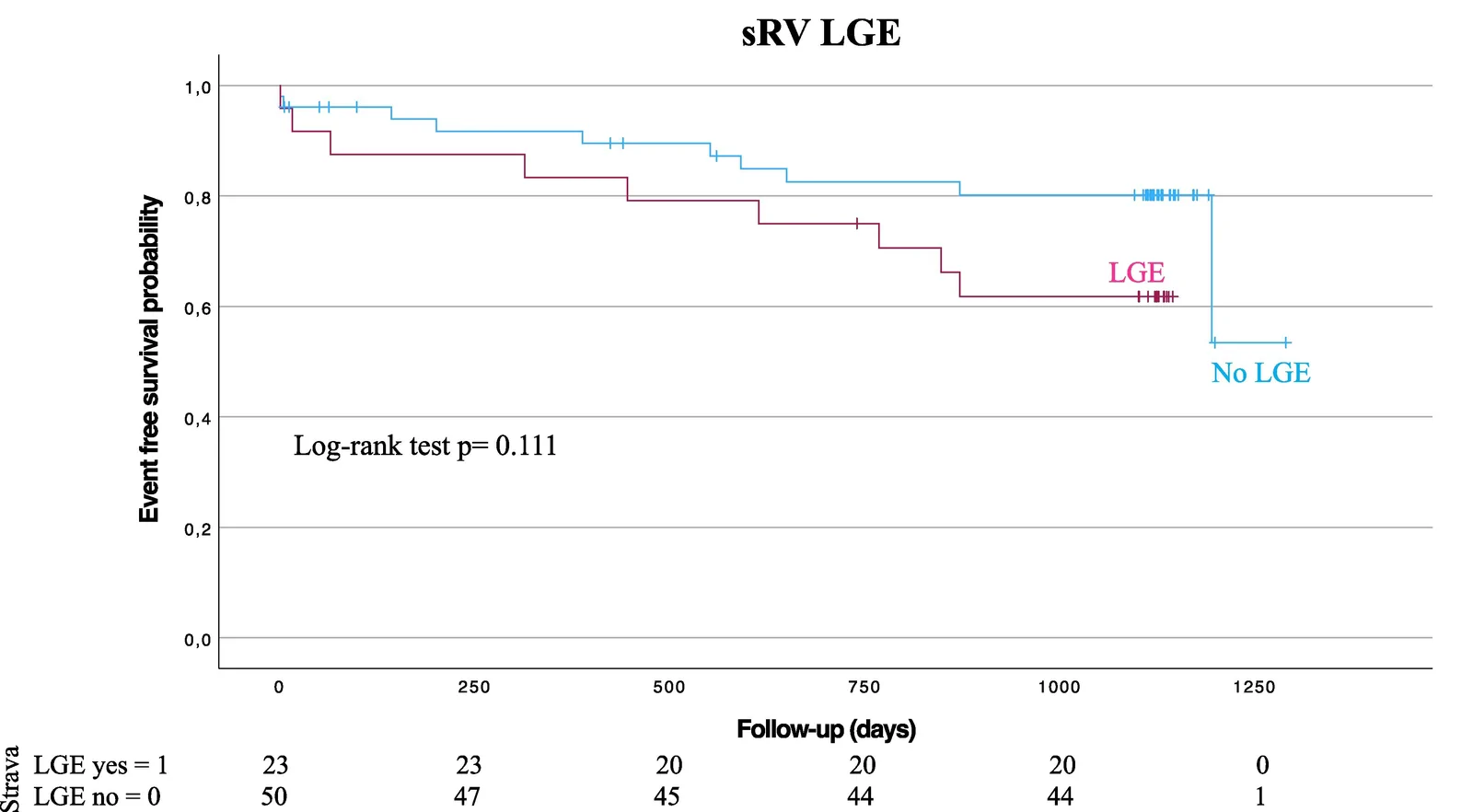
Systemic right ventricular late gadolinium enhancement (LGE) for prediction of outcome. Kaplan–Meier analysis showed no significant association between the presence of LGE and worse clinical outcome.

### Multivariate analyses and clinical outcome

Right ventricular (RV) basal mean native T1, RV mid-ventricular mean ECV, and the presence of LGE were analysed using Cox regression with regard to the clinical composite endpoint. As RVEF was also related to the outcome (see Supplementary material online, *Tables S8* and *S9*), it was additionally included in the analyses. Among all parameters, only RV basal mean native T1 remained independently associated with the composite outcome [RV mean T1: hazard ratio (HR) 4.9, 1.91; 21.2, *P* = 0.033; RV mid-ventricular mean ECV: HR 2.4 (0.6; 9.7), *P* = 0.238; RV LGE HR 1.0 (0.2; 4.4), *P* = 0.979; RVEF < 45%: HR 2.9 (0.7; 11.9), *P* = 0.144; the overall model was statistically significant, *P* = 0.009].

### Evolution of T1 mapping values and influence of treatment

*Table 2* provides an overview of the evolution of native T1 and ECV values for the basal and mid-ventricular levels for the whole RV and LV, respectively.

**Table 2:** Evolution of T1 mapping values

Two-way mixed ANOVA comparing T1 values between both groups and the different follow-up						
Right ventricle	Unit	Group	CMR baseline	CMR follow-up 1 year	CMR follow-up 3 years	*P* value
RV end-diastolic volume indexed	*mL/m^2^*	Placebo *n* = 31	117 ± 29	116 ± 28	119 ± 27	0.121
	*mL/m^2^*	Treatment *n* = 32	125 ± 27	129 ± 27	129 ± 33	
RV ejection fraction	*%*	Placebo *n* = 31	46 ± 6	46 ± 7	46 ± 7	0.256
	*%*	Treatment *n* = 32	45 ± 8	44 ± 8	44 ± 8	
**RV basal**						
Mean T1	*ms*	Placebo *n* = 20	1002 ± 11	1022 ± 10	1010 ± 8	0.163
	*ms*	Treatment *n* = 25	1021 ± 10	1029 ± 9	1043 ± 7	
Mean ECV		Placebo *n* = 14	0.291 ± 0.040	0.326 ± 0.079	0.287 ± 0.029	0.649
		Treatment *n* = 17	0.297 ± 0.038	0.355 ± 0.104	0.314 ± 0.039	
**RV mid-ventricular**						
Mean T1	*ms*	Placebo *n* = 21	1032 ± 49	1019 ± 53	1028 ± 55	0.968
	*ms*	Treatment *n* = 26	1035 ± 53	1024 ± 44	1037 ± 81	
Mean ECV		Placebo *n* = 17	0.301 ± 0.033	0.334 ± 0.072	0.306 ± 0.036	0.902
		Treatment *n* = 17	0.300 ± 0.034	0.350 ± 0.102	0.316 ± 0.056	

Left ventricle	Unit	Group	CMR baseline	CMR follow-up 1 year	CMR follow-up 3 years	*P* value
LV end-diastolic volume indexed	*mL/m^2^*	Placebo *n* = 31	75 ± 21	75 ± 23	78 ± 22	1.35
	*mL/m^2^*	Treatment *n* = 32	73 ± 17	75 ± 17	74 ± 19	
LV ejection fraction	*%*	Placebo *n* = 31	66 ± 5	67 ± 5	66 ± 5	0.018
	*%*	Treatment *n* = 32	65 ± 5	66 ± 5	65 ± 5	
**LV basal**						
Mean T1	*ms*	Placebo *n* = 20	1084 ± 97	1095 ± 77	1079 ± 116	0.201
	*ms*	Treatment *n* = 21	1081 ± 114	1071 ± 105	1110 ± 99	
Mean ECV		Placebo *n* = 14	0.398 ± 0.060	0.450 ± 0.116	0.384 ± 0.064	0.369
		Treatment *n* = 10	0.361 ± 0.044	0.450 ± 0.126	0.371 ± 0.049	
**LV mid-ventricular**						
Mean T1	*ms*	Placebo *n* = 21	1088 ± 115	1112 ± 112	1125 ± 115	0.968
	*ms*	Treatment *n* = 24	1063 ± 121	1094 ± 108	1098 ± 133	
Mean ECV		Placebo *n* = 16	0.397 ± 0.065	0.441 ± 0.116	0.398 ± 0.060	0.374
		Treatment *n* = 15	0.366 ± 0.065	0.463 ± 0.120	0.374 ± 0.060	

The treatment group showed a trend towards higher native T1 and ECV values at all study time points, but by mixed ANOVA statistics, no significant change of these parameters was noticed over the 3-year follow-up time. There was no influence of treatment on these parameters during follow-up (*Table 2*).

### Evolution of T1 mapping and correlations with functional parameters

As no impact of treatment on T1 and ECV in sRV patients was detected, patients from both the placebo and treatment groups were pooled together to compare T1 and ECV values to functional parameters and adverse outcomes. Evolution of pooled native T1 and ECV values over 1- and 3-year follow-up time are shown in Supplementary material online, *Table S3*, for the RV and Supplementary material online, *Table S4* for the LV.

Higher RV T1 and ECV values, defined as values above a given cut-off (75th percentile), tended to be associated with worse functional RV parameters, such as lower RV ejection fraction (RVEF), higher RV end-systolic volume indexed (RVESVi), higher RV end-diastolic volume indexed (RVEDVi), and higher RV mass, although these correlations were weak (see *Table 3*). A decline in functional parameters tended to correlate to higher RV T1 and ECV values, and some reached statistical significance (see Supplementary material online, *Tables S5* and S6).

**Table 3:** T1 and ECV relationship with functional parameters

Independent *t*-test comparing T1 values above vs. below the calculated 75th percentile for each functional parameter			
RV total T1 basal at baseline, P75	>1050 ms	<1050 ms	*P* value
Ejection fraction at baseline	43 ± 3 (*n* = 15)	47 ± 0.9 (*n* = 45)	0.067
Ejection fraction at follow-up 2	42 ± 3 (*n* = 13)	46 ± 0.9 (*n* = 40)	0.071
End-diastolic volume indexed at baseline	125 ± 34 (*n* = 15)	117 ± 27 (*n* = 45)	0.359
End-diastolic volume indexed at follow-up 2	140 ± 11 (*n* = 13)	121 ± 4 (*n* = 40)	0.054
End-systolic volume indexed at baseline	74 ± 8 (*n* = 15)	63 ± 3 (*n* = 45)	0.124
End-systolic volume indexed at follow-up 2	84 ± 40 (*n* = 13)	66 ± 20 (*n* = 40)	0.039
RV mass at baseline	94 ± 7 (*n* = 15)	90 ± 3 (*n* = 45)	0.572
RV mass at follow-up 2	95 ± 7 (*n* = 13)	95 ± 4 (*n* = 40)	0.976

RV total T1 basal at follow-up 2, P75	>1056 ms	<1056 ms	*P* value
Ejection fraction at follow-up 2	41 ± 10 (*n* = 12)	46 ± 6 (*n* = 41)	0.022
End-diastolic volume indexed at follow-up 2	137 ± 48 (*n* = 12)	121 ± 23 (*n* = 41)	0.122
End-systolic volume indexed at follow-up 2	83 ± 39 (*n* = 12)	66 ± 19 (*n* = 41)	0.047
RV mass at follow-up 2	103 ± 25 (*n* = 12)	92 ± 21 (*n* = 41)	0.118

RV total T1 mid-ventricular at baseline, P75	>1071 ms	<1071 ms	*P* value
Ejection fraction at baseline	42 ± 2 (*n* = 17)	47 ± 0.9 (*n* = 43)	0.046
Ejection fraction at follow-up 2	42 ± 2 (*n* = 15)	46 ± 1 (*n* = 38)	0.053
End-diastolic volume indexed at baseline	119 ± 27 (*n* = 30)	123 ± 35 (*n* = 31)	0.561
End-diastolic volume indexed at follow-up 2	136 ± 10 (*n* = 15)	121 ± 4 (*n* = 38)	0.110
End-systolic volume indexed at baseline	73 ± 7 (*n* = 17)	63 ± 3 (*n* = 43)	0.158
End-systolic volume indexed at follow-up 2	82 ± 9 (*n* = 15)	66 ± 3 (*n* = 38)	0.058
RV mass at baseline	96 ± 5 (*n* = 17)	86 ± 3 (*n* = 43)	0.104
RV mass at follow-up 2	103 ± 5 (*n* = 15)	89 ± 3 (*n* = 38)	0.040

RV total T1 mid-ventricular at CMR follow-up 2, P75	>1075 ms	<1075 ms	*P* value
Ejection fraction at follow-up 2	43 ± 9 (*n* = 15)	45 ± 7 (*n* = 44)	0.427
End-diastolic volume indexed at follow-up 2	136 ± 29 (*n* = 15)	122 ± 31 (*n* = 44)	0.143
End-systolic volume indexed at follow-up 2	79 ± 26 (*n* = 15)	69 ± 26 (*n* = 44)	0.206
RV mass at follow-up 2	96 ± 22 (*n* = 15)	92 ± 23 (*n* = 44)	0.533

RV total basal measured ECV at baseline, P75	>31%	<31%	*P* value
Ejection fraction at baseline	47 ± 8 (*n* = 13)	46 ± 7 (*n* = 38)	0.813
Ejection fraction at follow-up 2	43 ± 2 (*n* = 10)	46 ± 1 (*n* = 37)	0.337
End-diastolic volume indexed at baseline	124 ± 7 (*n* = 13)	118 ± 5 (*n* = 38)	0.494
End-diastolic volume indexed at follow-up 2	140 ± 9 (*n* = 10)	123 ± 5 (*n* = 37)	0.143
End-systolic volume indexed at baseline	67 ± 6 (*n* = 13)	65 ± 4 (*n* = 38)	0.783
End-systolic volume indexed at follow-up 2	80 ± 7 (*n* = 10)	68 ± 5 (*n* = 37)	0.239
RV mass at baseline	84 ± 6 (*n* = 13)	90 ± 4 (*n* = 38)	0.348
RV mass at follow-up 2	95 ± 7 (*n* = 10)	93 ± 4 (*n* = 37)	0.765

RV total basal measured ECV at CMR follow-up 2, P75	>31.9%	<31.9%	*P* value
Ejection fraction at follow-up 2	44 ± 9 (*n* = 11)	46 ± 7 (*n* = 35)	0.543
End-diastolic volume indexed at follow-up 2	126 ± 42 (*n* = 11)	120 ± 26 (*n* = 35)	0.564
End-systolic volume indexed at follow-up 2	73 ± 38 (*n* = 11)	66 ± 21 (*n* = 35)	0.416
RV mass at follow-up 2	94 ± 22 (*n* = 11)	91 ± 22 (*n* = 35)	0.726

RV total mid-ventricular measured ECV at baseline, P75	>33%	<33%	*P* value
Ejection fraction at baseline	47 ± 2 (*n* = 13)	46 ± 1 (*n* = 38)	0.664
Ejection fraction at follow-up 2	42 ± 3 (*n* = 8)	46 ± 1 (*n* = 38)	0.228
End-diastolic volume indexed at baseline	116 ± 7 (*n* = 13)	119 ± 5 (*n* = 38)	0.749
End-diastolic volume indexed at follow-up 2	135 ± 9 (*n* = 8)	123 ± 5 (*n* = 38)	0.332
End-systolic volume indexed at baseline	63 ± 6 (*n* = 13)	66 ± 4 (*n* = 38)	0.674
End-systolic volume indexed at follow-up 2	79 ± 9 (*n* = 8)	69 ± 4 (*n* = 38)	0.326
RV mass at baseline	80 ± 4 (*n* = 13)	90 ± 4 (*n* = 38)	0.140
RV mass at follow-up 2	89 ± 6 (*n* = 8)	93 ± 4 (*n* = 38)	0.680

RV total basal measured ECV at CMR follow-up 2, P75	>32.6%	<32.6%	*P* value
Ejection fraction at follow-up 2	44 ± 9 (*n* = 13)	46 ± 6 (*n* = 39)	0.585
End-diastolic volume indexed at follow-up 2	128 ± 39 (*n* = 13)	121 ± 25 (*n* = 39)	0.403
End-systolic volume indexed at follow-up 2	74 ± 36 (*n* = 13)	66 ± 20 (*n* = 39)	0.337
RV mass at follow-up 2	92 ± 23 (*n* = 13)	90 ± 21 (*n* = 39)	0.824

When using an RVEF cut-off of 45%, altered native T1 values were present in 23% of patients at the basal level and 37% at the mid-ventricular level among patients with preserved RVEF (*P* = 0.053 and *P* = 0.276). Patients with a decrease in RVEF of ≥5% did not show significantly higher baseline T1 mapping or ECV values (see Supplementary material online, *Table S7*).

### Inter- and intra-observer variability

Bland–Altman plots revealed excellent intra- and inter-observer variability for the RV and good variability for the LV T1 and ECV values (see Supplementary material online, *Figure S1*).

## Discussion

### T1 values and outcome

This prospective study shows that T1 mapping parameters help to predict clinical outcome in patients with a sRV (*Graphical abstract*).

This is in line with the results from Broberg *et al.*^22^ that demonstrated that higher ECV values were associated with increased risk of heart failure and arrhythmia in TGA patients with a sRV despite no association with other cardiac functional parameters. In contrast to Broberg *et al.*, the current study shows that not only ECV but also native T1 values are associated with clinical outcomes in patients with sRV. Native T1 values can easily be obtained in routine CMR exams, whereas determination of ECV requires administration of a contrast agent. Given that patients with sRV require regular CMR examinations, the possibility of identifying a biomarker predicting outcome without the need for contrast injection is particularly relevant and may help simplifying their follow-up. Moreover, it helps to shorten CMR exam time and could increase patients’ acceptance to undergo a CMR exam without contrast injection. In addition, new artificial intelligence techniques may further boost the information derived from the native T1 mapping and avoid the use of gadolinium-based contrast agents.^23^

Our results add new evidence on the role of native T1 in different cardiomyopathies. Puntmann *et al.*^24^ demonstrated that native T1 significantly predicts all-cause mortality in patients with non-ischaemic dilated cardiomyopathy, with a central role for native T1 as an independent predictor by multivariate analysis. Raisi-Estabragh *et al.*^25^ demonstrated that native T1 mapping was particularly good at predicting outcome in patients with non-ischaemic cardiomyopathies.

Our study underlines the importance of CMR T1 mapping in the evaluation of patients with complex CHD. We therefore believe that native T1 mapping parameters should be routinely performed and reported which should help to improve risk stratification in this population.

### The role of late gadolinium enhancement

Late gadolinium enhancement is the gold standard for the identification of macroscopic fibrosis in ischaemic and non-ischaemic cardiomyopathies, and an increasing number of studies demonstrated its prognostic value in non-ischaemic cardiomyopathies.^26–29^ Several studies have shown that LGE is related to reduced RV and LV functional parameters and outcome in CHD.^30^ The amount of LGE was related to ventricular function^31^ and particularly to the risk for atrial arrhythmias^32^ or reduced systolic and diastolic sRV function.^32^ However, in our study, similar to the findings of Broberg *et al.*,^22^ LGE was associated with reduced RV functional parameters but not significantly related to clinical outcome. In the present study, LGE showed a trend toward an association with the composite outcome, however, this did not reach statistical significance. A larger sample size and/or longer follow-up time might would have permitted showing a significant relationship between LGE and clinical outcome.

Of note, in the current study, almost all patients had insertion point fibrosis that was excluded from the analysis.

### T1 mapping values over time, influence of treatment, and relationship to right ventricular functional parameters

We hypothesized that the development of interstitial fibrosis detected by T1 mapping precedes the decline in function and exercise capacity and could be prevented by treatment with the PDE-5 inhibitor tadalafil. However, we could not show any differences between the treatment and placebo groups. This is consistent with the results of the main study, which did not demonstrate a benefit of tadalafil on RV function, RV end-systolic volume, or clinical endpoints.^21^ In line with the main SERVE study, a longer follow-up would probably not have changed our results.

We did not observe a statistically significant increase of T1 and ECV values during the 3-year follow-up, neither in the placebo nor in the treatment or the pooled group.

However, when dividing patients into groups with RV native T1 and ECV below versus above the 75th percentile, we found that higher RV native T1 and ECV values tended to be associated with lower sRV functional parameters, although the correlations were weak and not statistically significant for all parameters. These observations are in line with the hypothesis that interstitial fibrosis develops over time and has a negative impact on sRV functional parameters. Although there were significantly more adverse events in the group with RVEF < 45% at follow-up 2, we were not able to demonstrate in our study that the development of fibrosis precedes the decline in RVEF. This process is likely very subtle and slowly evolving, as also suggested in the recently published study of Kochav *et al* showing that RV functional parameters remain relatively stable over a long period of time in most patients.^33^ This might also explain why previous studies, often retrospective and smaller in size, showed conflicting results. While some studies demonstrated a relationship between native T1 mapping and impaired RV and LV functional parameters,^34,35^ the studies of Al-Wakeel-Marquard *et al.* and Shehu *et al.*^2,35^ did not observe such a relationship. Studies with longer follow-ups are warranted to confirm such correlations.

### Limitations

Power calculations for the primary endpoint in the SERVE study solidly determined sample size,^1^ but unfortunately, in addition to the usual drop-outs, T1 mapping as used for this sub-analysis was not performed in all patients, thus reducing the sample size of the present analysis. Moreover, some segments had to be excluded because of inadequate image quality or ventricular wall thickness.

It is difficult to define ‘normal’ values for sRV. We therefore chose the 75th percentile as it is a common cut-off point and divided the values in groups of acceptable size to be analysed.^36^

Fibrosis is not uniformly distributed along the longitudinal axis of the ventricles; variations in T1 and ECV values according to the myocardial region have been previously described. Native T1 values may be more influenced by diffuse tissue changes, whereas ECV is more sensitive to extracellular matrix expansion.^37^ Of note, basal measurements are more susceptible to partial volume effects, through-plane motion, and blood pool contamination, and these potential biases may be more pronounced in the RV, which has thinner ventricular walls. These factors may explain why mid-ventricular ECV showed significant associations with outcome, while mid-ventricular native T1 did not, and why basal native T1 but not basal ECV was associated with the outcome. T1 and ECV are two complementary parameters as they reflect overlapping but different tissue characteristics, showing that both parameters are relevant. Further studies may help to determine whether T1 or ECV is more appropriate for specific myocardial regions.

The inclusion of additional parameters, such as strain analysis, would have allowed a more comprehensive assessment of ventricular function. However, our study was designed to evaluate the incremental value, clinical relevance, and feasibility of T1 mapping parameters in our patient population. These parameters are readily available, particularly native T1 as it does not require contrast administration. Future studies incorporating more advanced functional imaging techniques, as well as direct comparisons between methods, would be valuable to further clarify their respective roles and complementary value.

The multi-centre design of our study is inherently associated with a potential influence of the use of different CMR scanners and vendors. However, the majority of CMR exams were performed on scanners from the same vendor. All images were analysed using the same post-processing software. One could have expected that ECV would better predict outcome than native T1, as its calculation is less dependent from the CMR scanner characteristics. It is therefore an even more remarkable and interesting observation that native T1 appears to better predict outcome in these patients than ECV.

## Conclusions

As one of the rare prospective studies in this field, our study shows that T1 mapping and in particular native T1 values, as non-invasive markers of myocardial fibrosis, can help to predict outcome in patients with sRV. We believe that this easily obtainable CMR biomarker, which does not require the use of contrast agents, should therefore be incorporated into routine clinical CMR imaging protocols to help improving risk stratification in sRV patients.

Our study did not allow the identification of an effective heart failure therapy in this population, nor could it establish a strong correlation between the evolution of T1 mapping parameters and functional CMR parameters over time. Further studies in this field are needed.

## Supplementary Material

oeag138_Supplementary_Data

## Data Availability

The data underlying this article are available in the article and in its online Supplementary material.
