# Supplementary material for: Cardiac magnetic resonance T1 mapping predicts outcome in patients with a systemic right ventricle: a sub-analysis of the SERVE study
Source: Eur Heart J Open. 2026 Sep 8;6(5):oeag138. doi: 10.1093/ehjopen/oeag138 (PMC13649081; doi:10.1093/ehjopen/oeag138)

Supplemental appendix Figures:

Supplemental Figure S1: Bland-altmann plots for intra- and inter-observer variability

Intra- and inter-observer variability RV mean total T1

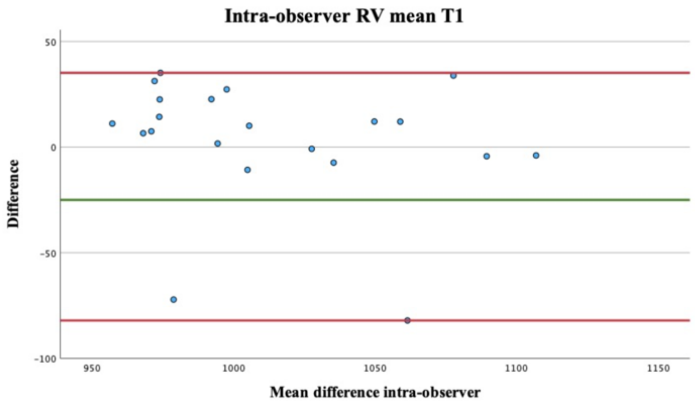

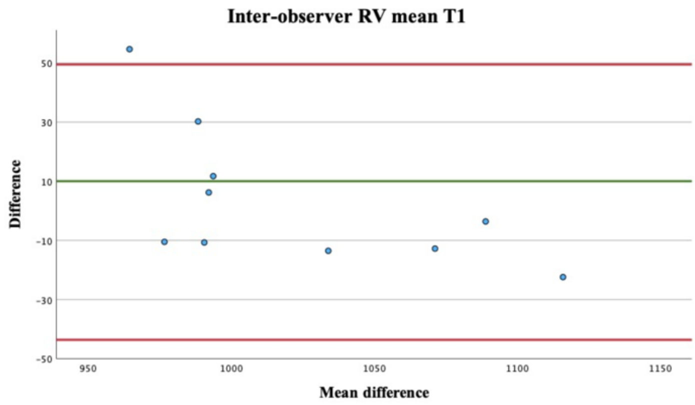

Intra- and inter-observer variability LV mean total T1

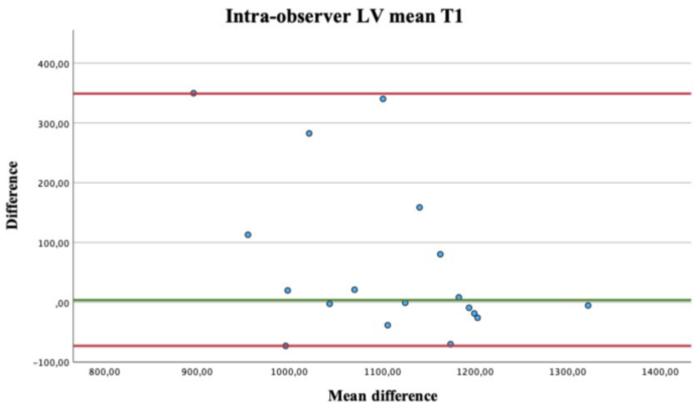

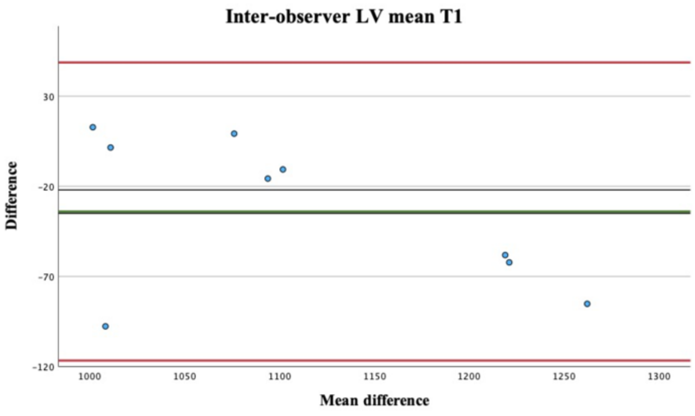

Intra- and inter-observer variability RV mean total ECV

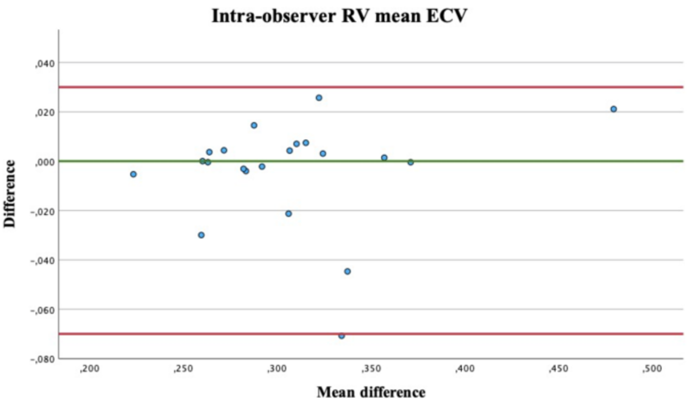

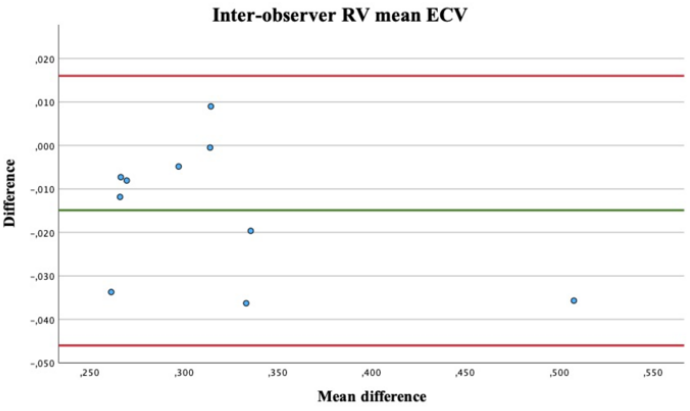

Intra- and inter-observer variability LV mean total ECV

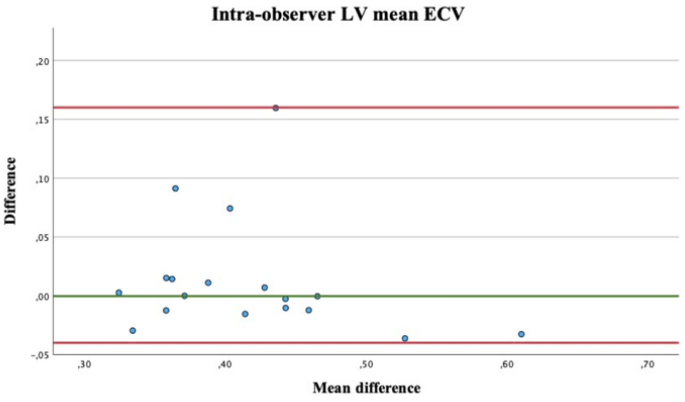

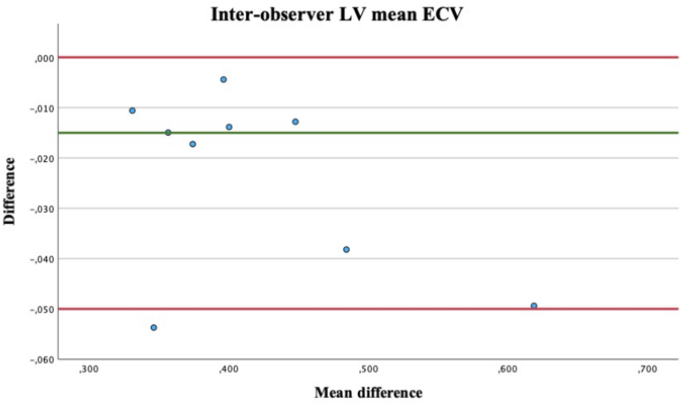

Supplement: Supplementary file 1 [file oeag138_supplementary_data.zip › Supplemental_Appendix_Figures_revised_.pdf]
