# Supplementary material for: Cardiac magnetic resonance T1 mapping predicts outcome in patients with a systemic right ventricle: a sub-analysis of the SERVE study
Source: Eur Heart J Open. 2026 Sep 8;6(5):oeag138. doi: 10.1093/ehjopen/oeag138 (PMC13649081; doi:10.1093/ehjopen/oeag138)
Supplement: Supplementary file 1 [file oeag138_supplementary_data.zip › Supplemental_Appendix_Tables_revised.docx]

**Supplemental appendix Tables:**

[**Supplemental Table S1:** T1 mapping and ECV for outcome prediction	2](#_Toc230426698)

[**Supplemental Table S2:** LGE for outcome prediction	3](#_Toc230426699)

[**Supplemental Table S3:** Right ventricle T1 and ECV values pooled (Placebo and treatment)	4](#_Toc230426700)

[**Supplemental Table S4:** Left ventricle T1 and ECV values pooled (Placebo and treatment)	6](#_Toc230426701)

[**Supplemental Table S5:** Linear regressions:	7](#_Toc230426702)

[**Supplemental table S6:** Comparison of patients with altered T1 mapping values *(P75 T1 basal at baseline)* and altered or preserved RVEF	12](#_Toc230426703)

[**Supplemental table S7**: Difference between mean T1 and ECV values between groups with ≥5% RVEF reduction at follow-up2 compared to baseline RVEF	13](#_Toc230426704)

[**Supplemental table S8:** Difference in the incidence of adverse events between group with preserved versus reduced RVEF at baseline	14](#_Toc230426705)

[**Supplemental table S9**: Difference in functional parameters between group with adverse events versus no adverse events	15](#_Toc230426706)

| **Supplemental Table S1:** T1 mapping and ECV for outcome prediction | | | |
| --- | --- | --- | --- |
| **Basal sRV Baseline** | | | |
|  | Adverse event | No adverse event | p-value |
|  | N = 14 | N = 46 |  |
| Native mean T1 | 1048.1 ± 81.8 | 1011.5 ± 46.2 | 0.037 |
|  | N=11 | N=40 |  |
| Mean ECV | 0.307 ± 0.059 | 0.292 ± 0.039 | 0.310 |
|  |  |  |  |
| **Mid-ventricular sRV Baseline** | | | |
|  | N = 13 | N = 47 |  |
| Native mean T1 | 1049.1 ± 50.7 | 1033.7 ± 63.9 | 0.426 |
|  | N=11 | N=40 |  |
| Mean ECV | 0.319 ± 0.038 | 0.304 ± 0.035 | 0.209 |
|  |  |  |  |
| **Basal sRV follow-up2** | | | |
|  | N = 11 | N = 44 |  |
| Native mean T1 | 1034.5 ± 43.6 | 1029.2 ± 36.7 | 0.682 |
|  | N=8 | N=39 |  |
| Mean ECV | 0.294 ± 0.019 | 0.300 ± 0.037 | 0.591 |
|  |  |  |  |
| **Mid-ventricular sRV follow-up2** | | | |
|  | N = 12 | N = 49 |  |
| Native mean T1 | 1069.4 ± 56.8 | 1018.0 ± 74.5 | 0.029 |
|  | N = 9 | N = 44 |  |
| Mean ECV | 0.319 ± 0.025 | 0.305 ± 0.046 | 0.389 |

| **Supplemental Table S2:** LGE for outcome prediction | | | |
| --- | --- | --- | --- |
|  | Adverse event | No adverse event | p-value |
| Presence of LGE | 9 | 15 | 0.097 |
| No LGE | 10 | 41 |  |

| **Supplemental Table S3:** Right ventricle T1 and ECV values pooled (Placebo and treatment) | | | | |
| --- | --- | --- | --- | --- |
| *Repeated measures ANOVA comparing T1 values during follow-up* | | | | |
| **RV functional parameters** | CMR **Baseline** | CMR **follow-up 1-year** | CMR **follow-up 3-year** | **p-value** |
| Age, years | 39 (25-75) |  |  |  |
| Women, % | 33 |  |  |  |
| Tadalafil, n | 30 | 30 | 31 |  |
| Placebo, n | 31 | 32 | 30 |  |
|  |  |  |  |  |
| Weight, kg | 77 ± 14 (n=84) | 77 ± 13 (n=84) | 78 ± 14 (n=84) | 0.239 |
| Height, cm | 173 ± 8 (n=84) | 173 ± 8 (n=84) | 173 ± 8 (n=84) | 0.945 |
| BMI, kg/m^2^ | 26 ± 4 (n=84) | 26 ± 4 (n=84) | 26 ± 4 (n=84) | 0.221 |
|  |  |  |  |  |
| BSA, m^2^ | 1.9 ± 0.18 (n=63) | 1.9 ± 0.18 (n=63) | 1.9 ± 0.19 (n=63) | 0.255 |
| End-diastolic volume, ml | 230 ± 58 (n=63) | 234 ± 62 (n=63) | 238 ± 65 (n=63) | 0.009 |
| End-diastolic volume indexed, ml/m^2^ | 121 ± 28 (n=63) | 123 ± 28 (n=63) | 124 ± 30 (n=63) | 0.064 |
| End-systolic volume indexed, ml/m^2^ | 67 ± 23 (n=63) | 68 ± 24 (n=63) | 70 ± 26 (n=63) | 0.009 |
| RV Mass, g/m^2^ | 90 ± 22 (n=63) | 92 ± 22 (n=63) | 93 ± 22 (n=63) | 0.018 |
|  |  |  |  |  |
| RV Ejection fraction, % | 45 ± 7 (n=63) | 45 ± 7 (n=63) | 45 ± 7 (n=63) | 0.057 |
|  |  |  |  |  |
| Hematocrit, % | 44 ± 3 (n=62) | 43 ± 4 (n=62) | 44 ± 4 (n=62) | 0.040 |
|  |  |  |  |  |
| **RV pre contrast**  **Basal** | CMR **Baseline** | CMR **follow-up 1-year** | CMR **follow-up 3-year** | **p-value** |
| Mean total T1, ms | 1013 ± 50 (n=45) | 1026 ± 43 (n=45) | 1029 ± 38 (n=45) | 0.045 |
|  |  |  |  |  |
| Blood, ms | 1516 ± 86 (n=45) | 1512 ± 88 (n=45) | 1525 ± 84 (n=45) | 0.358 |
|  |  |  |  |  |
| ECV mean total, % | 29.4 ± 3.8 (n=31) | 34.2 ± 9.3 (n=31) | 30.2 ± 3.7 (n=31) | 0.010 |
|  |  |  |  |  |
| **RV pre contrast**  **Mid-ventricular** | CMR **Baseline** | CMR **follow-up 1-year** | CMR **follow-up 3-year** | **p-value** |
| Mean total T1, ms | 1034 ± 51 (n=47) | 1022 ± 47 (n=47) | 1033 ± 70 (n=47) | 0.428 |
|  |  |  |  |  |
| Blood, ms | 1518 ± 88 (n=47) | 1509 ± 88 (n=47) | 1515 ± 87 (n=47) | 0.639 |
|  |  |  |  |  |
| ECV mean total, % | 30.1 ± 3.3 (n=34) | 34.5 ± 8.7 (n=34) | 31.1 ± 4.7 (n=34) | 0.014 |

| **Supplemental Table S4:** Left ventricle T1 and ECV values pooled (Placebo and treatment) | | | | |
| --- | --- | --- | --- | --- |
| *Repeated measures ANOVA comparing T1 values during follow-up* | | | | |
| **LV functional parameters** | CMR **Baseline** | CMR **follow-up 1-year** | CMR **follow-up 3-year** | **p-value** |
| End-diastolic volume, ml | 139 ± 34 (n=63) | 142 ± 37 (n=63) | 145 ± 37 (n=63) | 0.033 |
| End-diastolic volume indexed, ml/m^2^ | 74 ± 19 (n=63) | 75 ± 20 (n=63) | 76 ± 20 (n=63) | 0.118 |
| End-systolic volume indexed, ml/m^2^ | 25 ± 9 (n=63) | 26 ± 9 (n=63) | 26 ± 9 (n=63) | 0.160 |
| LV Ejection fraction, % | 66 ± 5 (n=63) | 66 ± 5 (n=63) | 66 ± 5 (n=63) | 0.296 |
|  |  |  |  |  |
| **LV pre contrast**  **Basal** | **CMR Baseline** | **CMR follow-up 1-year** | **CMR follow-up 3-year** | **p-value** |
| Mean total T1, ms | 1083 ± 105 (n=41) | 1082 ± 92 (n=41) | 1095 ± 108 (n=41) | 0.626 |
|  |  |  |  |  |
| Blood, ms | 1489 ± 89 (n=41) | 1481 ± 92 (n=41) | 1493 ± 88 (n=41) | 0.545 |
|  |  |  |  |  |
| ECV mean total, % | 38.2 ± 5.5 (n=24) | 45.0 ± 11.8 (n=24) | 37.9 ± 5.7 (n=24) | 0.008 |
|  |  |  |  |  |
| **LV pre contrast Mid-ventricular** | CMR **Baseline** | CMR **follow-up 1-year** | CMR **follow-up 3-year** | **p-value** |
| Mean total T1, ms | 1075 ± 118 (n=45) | 1103 ± 109 (n=45) | 1111 ± 124 (n=45) | 0.085 |
|  |  |  |  |  |
| Blood, ms | 1475 ± 85 (n=44) | 1474 ± 89 (n=44) | 1489 ± 80 (n=44) | 0.274 |
|  |  |  |  |  |
| ECV mean total, % | 38.2 ± 6.6 (n=31) | 45.2 ± 11.7 (n=31) | 38.6 ± 6.0 (n=31) | 0.009 |

| **Supplemental Table S5:** Linear regressions: | | | | | |
| --- | --- | --- | --- | --- | --- |
| **RV** | **R** | **R square** | **adjusted R square** | **p-value** | **Pearson’s correlation** |
| RVEF at CMR follow-up2 by T1 total basal at Baseline | 0.086 | 0.007 | -0.012 | 0.539 |  |
| RVEF at CMR follow-up2 by T1 total mid-ventricular at Baseline | 0.012 | 0.000 | -0.019 | 0.930 |  |
| RVEF at CMR follow-up2 by measured total basal ECV at Baseline | 0.290 | 0.084 | 0.063 | 0.048 | -0.290 |
| RVEF at CMR follow-up2 by measured total mid-ventricular ECV at Baseline | 0.154 | 0.024 | 0.001 | 0.313 |  |
|  |  |  |  |  |  |
| RVEF at CMR baseline by T1 total basal at follow-up 2 | 0.251 | 0.063 | 0.045 | 0.065 |  |
| RVEF at CMR follow-up 2 by T1 total basal at follow-up 2 | 0.307 | 0.094 | 0.077 | 0.025 | -0.307 |
| RVEF at CMR baseline by T1 total mid-ventricular at follow-up 2 | 0.313 | 0.098 | 0.082 | 0.014 | -0.313 |
| RVEF at CMR follow-up 2 by T1 total mid-ventricular at follow-up 2 | 0.314 | 0.098 | 0.083 | 0.016 | -0.314 |
|  |  |  |  |  |  |
| RVEF at CMR baseline by total basal measured ECV at follow-up 2 | 0.175 | 0.031 | 0.009 | 0.239 |  |
| RVEF at CMR follow-up 2 by total basal measured ECV at follow-up 2 | 0.258 | 0.067 | 0.046 | 0.083 |  |
| RVEF at CMR baseline by total mid-ventricular measured ECV at follow-up 2 | 0.070 | 0.005 | -0.015 | 0.619 |  |
| RVEF at CMR follow-up 2 by total mid-ventricular measured ECV at follow-up 2 | 0.085 | 0.007 | -0.013 | 0.550 |  |
|  |  |  |  |  |  |
| RV Enddiastolic volume indexed at follow-up 2 by T1 total basal at CMR Baseline | 0.181 | 0.033 | 0.014 | 0.194 |  |
| RV Enddiastolic volume indexed at follow-up 2 by T1 total mid-ventricular at CMR Baseline | 0.029 | 0.001 | -0.019 | 0.834 |  |
| RV Enddiastolic volume indexed at follow-up 2 by basal measured ECV total at CMR Baseline | 0.352 | 0.124 | 0.105 | 0.015 | 0.352 |
| RV Enddiastolic volume indexed at follow-up 2 by measured ECV total at CMR Baseline | 0.069 | 0.005 | -0.018 | 0.654 |  |
|  |  |  |  |  |  |
| RV Endsystolic volume indexed at follow-up 2 by  T1 total basal at CMR Baseline | 0.286 | 0.082 | 0.055 | 0.091 |  |
| RV Endsystolic volume indexed at follow-up 2 by T1 total mid-ventricular at CMR Baseline | 0.103 | 0.011 | -0.019 | 0.557 |  |
| RV Endsystolic volume indexed at follow-up 2 by measured total ECV basal at CMR Baseline | 0.177 | 0.031 | -0.005 | 0.358 |  |
| RV Endsystolic volume indexed at follow-up 2 by mid-ventricular total measured ECV at CMR Baseline | 0.466 | 0.217 | 0.187 | 0.012 | 0.466 |

| **RV** | **R** | **R square** | **adjusted R square** | **p-value** | **Pearson’s correlation** |
| --- | --- | --- | --- | --- | --- |
| RVEF at Baseline versus basal total T1 at Baseline | 0.078 | 0.006 | -0.011 | 0.555 |  |
| RVEF at Baseline versus mid-ventricular total T1 at Baseline | 0.056 | 0.003 | -0.014 | 0.673 |  |
| RVEF at Baseline versus basal total measured ECV at Baseline | 0.151 | 0.023 | 0.003 | 0.290 |  |
| RVEF at Baseline versus mid-ventricular total measured ECV at Baseline | 0.029 | 0.001 | -0.020 | 0.844 |  |
|  |  |  |  |  |  |
| Enddiastolic volume indexed at CMR baseline versus basal total T1 at CMR baseline | 0.039 | 0.002 | -0.016 | 0.767 |  |
| Enddiastolic volume indexed at CMR baseline versus mid-ventricular total T1 at CMR baseline | 0.061 | 0.004 | -0.013 | 0.642 |  |
| Enddiastolic volume indexed at CMR baseline versus basal total measured ECV at CMR baseline | 0.215 | 0.046 | 0.027 | 0.131 |  |
| Enddiastolic volume indexed at CMR baseline versus mid-ventricular total measured ECV at CMR baseline | 0.095 | 0.009 | -0.012 | 0.513 |  |
|  |  |  |  |  |  |
| Enddiastolic volume indexed at CMR baseline versus basal total T1 at CMR follow-up 2 | 0.149 | 0.022 | 0.004 | 0.277 |  |
| Enddiastolic volume indexed at CMR baseline versus mid-ventricular total T1 at CMR follow-up 2 | 0.186 | 0.035 | 0.018 | 0.151 |  |
| Enddiastolic volume indexed at CMR baseline versus basal total measured ECV at CMR follow-up 2 | 0.040 | 0.002 | -0.021 | 0.791 |  |
| Enddiastolic volume indexed at CMR baseline versus mid-ventricular total measured ECV at CMR follow-up 2 | 0.047 | 0.002 | -0.017 | 0.737 |  |
|  |  |  |  |  |  |
| Enddiastolic volume indexed at CMR follow-up 2 versus basal total T1 at CMR follow-up 2 | 0.187 | 0.035 | 0.016 | 0.181 |  |
| Enddiastolic volume indexed at CMR follow-up 2 versus mid-ventricular total T1 at CMR follow-up 2 | 0.165 | 0.027 | 0.010 | 0.211 |  |
| Enddiastolic volume indexed at CMR follow-up 2 versus basal total measured ECV at CMR follow-up 2 | 0.063 | 0.004 | -0.019 | 0.677 |  |
| Enddiastolic volume indexed at CMR follow-up 2 versus mid-ventricular total measured ECV at CMR follow-up 2 | 0.063 | 0.004 | -0.016 | 0.659 |  |
|  |  |  |  |  |  |
| Endsystolic volume indexed at CMR baseline versus basal total T1 at CMR baseline | 0.162 | 0.026 | 0.004 | 0.288 |  |
| Endsystolic volume indexed at CMR baseline versus mid-ventricular total T1 at CMR baseline | 0.063 | 0.004 | -0.020 | 0.685 |  |
| Endsystolic volume indexed at CMR baseline versus basal total measured ECV at CMR baseline | 0.043 | 0.002 | -0.026 | 0.797 |  |
| Endsystolic volume indexed at CMR baseline versus mid-ventricular total measured ECV at CMR baseline | 0.164 | 0.027 | -0.001 | 0.333 |  |
|  |  |  |  |  |  |
| Endsystolic volume indexed at CMR baseline versus basal total T1 at CMR follow-up 2 | 0.090 | 0.008 | -0.018 | 0.582 |  |
| Endsystolic volume indexed at CMR baseline versus mid-ventricular total T1 at CMR follow-up 2 | 0.193 | 0.037 | 0.015 | 0.198 |  |
| Endsystolic volume indexed at CMR baseline versus basal total measured ECV at CMR follow-up 2 | 0.049 | 0.002 | -0.029 | 0.785 |  |
| Endsystolic volume indexed at CMR baseline versus mid-ventricular total measured ECV at CMR follow-up 2 | 0.204 | 0.042 | 0.016 | 0.212 |  |
|  |  |  |  |  |  |
| Endsystolic volume indexed at CMR follow-up 2 versus basal total T1 at CMR follow-up 2 | 0.067 | 0.004 | -0.029 | 0.717 |  |
| Endsystolic volume indexed at CMR follow-up 2 versus mid-ventricular total T1 at CMR follow-up 2 | 0.239 | 0.057 | 0.030 | 0.154 |  |
| Endsystolic volume indexed at CMR follow-up 2 versus basal total measured ECV at CMR follow-up 2 | 0.445 | 0.198 | 0.165 | 0.023 | 0.445 |
| Endsystolic volume indexed at CMR follow-up 2 versus mid-ventricular total measured ECV at CMR follow-up 2 | 0.044 | 0.002 | -0.034 | 0.817 |  |

| **Supplemental table S6:** Comparison of patients with altered T1 mapping values *(P75 T1 basal at baseline)* and altered or preserved RVEF | | | | |
| --- | --- | --- | --- | --- |
| Total T1 basal at Baseline, **P75** | **≤ 1050 ms** | **> 1050 ms** |  | p-value |
| RVEF < 45% | 17 (74%) | 6 (26%) | 23 |  |
| RVEF ≥45% | 23 (77%) | 7 (23%) | 30 |  |
|  | 40 | 13 |  | 0.817 |
| Comparison of patients with altered T1 mapping values *(P75 T1 mid-ventricular at baseline)* and altered or preserved RVEF | | | | |
| Total T1 mid-ventricular at Baseline, **P75** | **≤ 1071 ms** | **> 1071 ms** |  | p-value |
| RVEF < 45% | 16 (67%) | 8 (33%) | 24 |  |
| RVEF ≥45% | 27 (63%) | 8 (37%) | 35 |  |
|  | 43 | 16 |  | 0.374 |
| Comparison of patients with altered ECV values *(P75 ECV basal at baseline)* and altered or preserved RVEF | | | | |
| ECV basal at Baseline, **P75** | **≤ 31%** | **> 31%** |  | p-value |
| RVEF < 45% | 10 (62%) | 6 (38%) | 16 |  |
| RVEF ≥45% | 21 (68%) | 10 (32%) | 31 |  |
|  | 31 | 16 |  | 0.719 |
| Comparison of patients with altered ECV values *(P75 ECV mid-ventricular at baseline)* and altered or preserved RVEF | | | | |
| ECV mid-ventricular at Baseline, **P75** | **≤ 33%** | **> 33%** |  | p-value |
| RVEF < 45% | 15 (75%) | 5 (25%) | 20 |  |
| RVEF ≥45% | 26 (79%) | 7 (7%) | 33 |  |
|  | 41 | 12 |  | 0.749 |
| Comparison of patients with altered T1 mapping values *(P75 T1 basal at follow-up2)* and altered or preserved RVEF | | | | |
| Total T1 basal at follow-up2, **P75** | **≤ 1056 ms** | **> 1056 ms** |  | p-value |
| RVEF < 45% | 16 (70%) | 7 (30%) | 23 |  |
| RVEF ≥45% | 25 (83%) | 5 (17%) | 30 |  |
|  | 41 | 12 |  | 0.235 |
| Comparison of patients with altered T1 mapping values *(P75 T1 mid-ventricular at follow-up2)* and altered or preserved RVEF | | | | |
| Total T1 mid-ventricular at follow-up2, **P75** | **≤ 1075 ms** | **> 1075 ms** |  | p-value |
| RVEF < 45% | 20 (77%) | 6 (23%) | 26 |  |
| RVEF ≥45% | 26 (26%) | 9 (9%) | 35 |  |
|  | 46 | 15 |  | 0.813 |

| **Supplemental table S7**: Difference between mean T1 and ECV values between groups with ≥5% RVEF reduction at follow-up2 compared to baseline RVEF | | | | |
| --- | --- | --- | --- | --- |
| Difference  **RVEF follow-up2 versus baseline** | | ≥5% | <5% | p-value |
| T1 basal baseline | *ms* | 987 ± 33 (N=5) | 1017 ± 53 (N=46) | 0.227 |
| ECV basal baseline | *%* | 29 ±5 (N=5) | 29 ± 4 (N=40) | 0.908 |
|  |  |  |  |  |
| T1 mid-ventricular baseline | *ms* | 1014 ± 58 (N=7) | 1040 ± 67 (N=44) | 0.335 |
| ECV mid-ventricular baseline | *%* | 27 (25-38) | 30 ± 3 | 0.114 |

| **Supplemental table S8:** Difference in the incidence of adverse events between group with preserved versus reduced RVEF at baseline | | | | |
| --- | --- | --- | --- | --- |
| **RVEF** at **baseline** | Adverse event : **NO** | Adverse event : **YES** |  | p-value |
| RVEF **<45%** | 23 (66%) | 12 (34%) | 35 |  |
| RVEF **≥45%** | 35 (81%) | 8 (19%) | 43 |  |
|  | 58 | 20 |  | 0.115 |
| Difference in the incidence of adverse events between group with preserved versus reduced RVEF at follow-up2 | | | | |
| **RVEF** at **follow-up2** | Adverse event : **NO** | Adverse event : **YES** |  | p-value |
| RVEF **< 45%** | 20 (67%) | 10 (33%) | 30 |  |
| RVEF **≥45%** | 29 (88%) | 4 (12%) | 33 |  |
|  | 49 | 14 |  | 0.043 |

| **Supplemental table S9**: Difference in functional parameters between group with adverse events versus no adverse events | | | | |
| --- | --- | --- | --- | --- |
| **Baseline** |  | Adverse event : **NO** | Adverse event : **YES** | p-value |
| RVEF | *%* | 46% ± 6 | 41% ± 9 | 0.033 |
| RV end-diastolic volume indexed | *ml/m^2^* | 116 (67-212) | 124 (87-244) | 0.107 |
| RV end-systolic volume indexed | *ml/m^2^* | 60 (36-135) | 71 (40-178) | 0.036 |
